# The endocrine role of hepatokines: implications for human health and disease

**DOI:** 10.3389/fendo.2025.1663353

**Published:** 2025-09-29

**Authors:** Pedro Iglesias

**Affiliations:** ^1^ Department of Endocrinology and Nutrition, Hospital Universitario Puerta de Hierro Majadahonda, Madrid, Spain; ^2^ Instituto de Investigación Sanitaria Puerta de Hierro Segovia de Arana, Majadahonda, Madrid, Spain

**Keywords:** hepatokines, metabolic diseases, insulin resistance, non-alcoholic fatty liver disease, inflammation, biomarkers, therapeutic targets, FGF21

## Abstract

The present narrative review analyzes the biology of hepatokines as well as their physiological functions and their effect on metabolism and different endocrine-metabolic diseases. Hepatokines are proteins secreted by the liver that play important roles in the regulation of energy homeostasis, inflammation and insulin resistance, behaving as relevant factors in the pathophysiology of pathologies such as obesity, non-alcoholic fatty liver disease (NAFLD) and type 2 diabetes. These include FGF21, fetuin-A, selenoprotein P, IGF-1, HGF, and ANGPTL family proteins. In addition, advances in therapies aimed at modulating the action of these proteins, such as FGF21 analogues and ANGPTL3 inhibitors, with good results in NASH resolution, improved insulin sensitivity and serum lipid reduction, are discussed. Molecular pathways related to hepatic signaling, including transcription factors and mechanisms regulating hepatic secretion, are also addressed, opening possibilities for innovative therapeutic strategies. Understanding hepatokines and their mechanisms promotes the development of personalized treatments for metabolic diseases, contributing to improve metabolic health and prevent related complications. This review highlights the importance of integrating liver biology with the clinic to address current challenges in the management of metabolic diseases.

## Introduction

The liver has traditionally been considered as an organ in charge of metabolism and with detoxification functions. However, in recent years its role as an endocrine organ has gained clinical relevance. Its ability to synthesize and release proteins that have the capacity to act at a distance in other organs and tissues such as adipose tissue, skeletal muscle, the central nervous system and the pancreas, reinforces its role in the regulation of energy and metabolism (1).

Like adipokines (from adipose tissue) (2, 3) and myokines (from skeletal muscle) (4, 5), hepatokines are released by the liver in a bioactive condition and are endocrine, paracrine and autocrine mediators that, at the systemic level, regulate metabolic, immunologic and proliferative functions (6–8). The hormonal, nutritional and stress stimuli finely regulate hepatokine synthesis and secretion. The presence of a dysfunction in these mechanisms has been related to the development of pathologies such as type 2 diabetes (9), non-alcoholic fatty liver disease (NAFLD) (10, 11) and various cardiovascular diseases (12).

The term hepatokine gained popularity in the early 21st century due to the establishment of proteins like fetuin-A, FGF21 and selenoprotein P (SeP). These proteins possessed activities outside of the liver and possessed well-characterized hormonal activities (13–15). From then onwards, there has been increasing scientific curiosity in this area with additional hepatokines identified and new functions emerging in various physiological and pathological states.

The goal of this review is to provide a new and critical overview of the hepatokines’ role in human disease and health. Their mechanisms of regulation, systemic physiological effects, involvement in typical diseases such as type 2 diabetes, obesity, metabolic-associated steatotic liver disease (MASLD), and cardiovascular disease, and potential use as biomarkers or therapeutic targets will be discussed.

## Biology of hepatokines

### Synthesis and release

Hepatokines are synthesized almost entirely in hepatocytes (4, 7). Other liver cells may also be implicated. The production is initiated through the activation of a few, specifically chosen genes whose transcription is subject to nutritional status, availability of many different circulating hormones and inflammatory mediators, as a deciding determinant of the process. The messenger RNAs, upon transcription, are translated to proteins in the endoplasmic reticulum. Here these proteins receive a three-dimensional conformation and are processed, such as through glycosylation. Protein production and packaging is via the Golgi apparatus. Upon completion of the process, these proteins are secreted in vesicles (4, 7, 8, 16, 17).

Regarding the release mechanisms, hepatokines follow two main pathways. the classical (conventional) secretory pathway depends on the endoplasmic reticulum-Golgi apparatus system and is associated with final exocytosis, as happens with proteins such as FGF21 or fetuin-A (7, 10). Instead, there are unconventional pathways through which some of the hepatokines are secreted through extracellular vesicles, such as exosomes, or through specific membrane channels (18, 19). The pathway is chosen depending on the type of hepatokine as well as the cellular and physiological environment.

### Regulation of secretion

**Figure 1:**
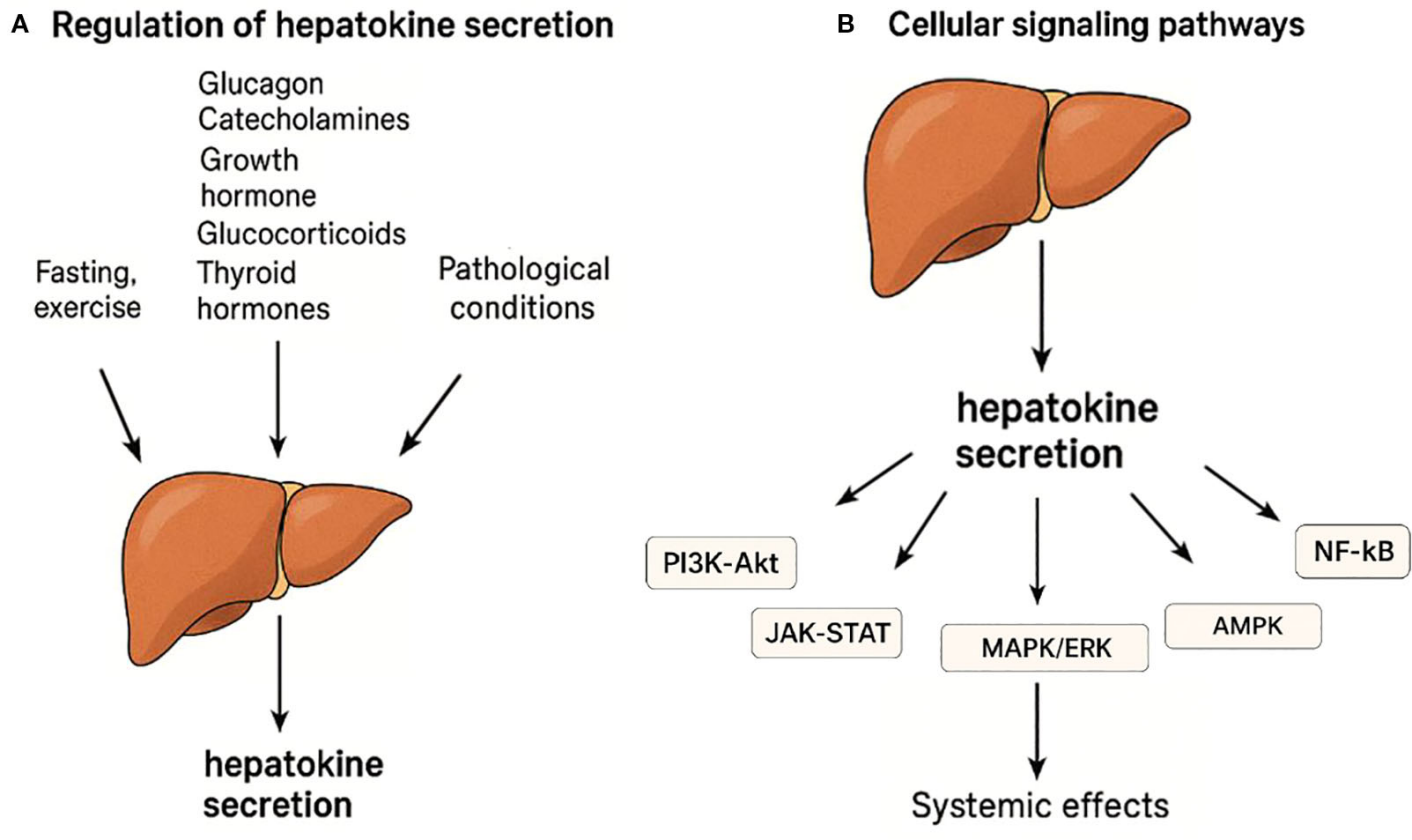
Regulation and signaling pathways of hepatokine secretion. **(A)** Hepatokine secretion is regulated by hormonal, metabolic, and physiological stimuli such as fasting, exercise, and pathological conditions. Hormones including glucagon, catecholamines, growth hormone, glucocorticoids, and thyroid hormones influence hepatokine expression. **(B)** Hepatokines act through major intracellular signaling pathways including PI3K-Akt, JAK-STAT, MAPK/ERK, AMPK, and NF-κB to exert systemic metabolic and inflammatory effects.

The production and release of hepatokines are strictly controlled by signals from stress, hormonal, metabolic and nutritional states (**Figure 1**). During fasting, hormones such as glucagon and catecholamines predominate to trigger the expression of hepatokines such as fibroblast growth factor 21 (FGF21) and angiopoietin-like 4 (ANGPTL4) through the activation of the nuclear receptor PPAR-α (20, 21). Physical exercise would also elicit the production of hepatokines with metabolically protective activity through the same pathway. The postprandial phase is controlled by insulin to suppress the expression of FGF21 and fetuin-A but could stimulate others such as IGFBP1, follistatin and even adropine (21).

Other hormones also regulate hepatokine secretion: glucocorticoids increase fetuin-A and ANGPTL4, particularly during fasting, by synergizing with glucagon to activate the gluconeogenic and hepatokine gene programs in hepatocytes (12); growth hormone (GH) increases IGF-1 and FGF21 expression via lipolysis-driven PPAR-α activation (22, 23); thyroid hormones increase FGF21 and SeP; while estrogens reduce fetuin-A and SeP (12).

This hormonal regulation is effected by transcriptional regulators such as PPARα, FOXO1 and SREBP-1c, and intracellular signaling such as PI3K/AKT, AMPK and NF-κB, which coordinate metabolic and inflammatory signals to control energy homeostasis and systemic response (21, 24, 25).

Under physiological conditions such as fasting or exercise, the release of hepatokines promotes metabolic adaptation (26). However, in pathological conditions such as type 2 diabetes or NAFLD, there is an excessive and dysregulated release of hepatokines with proinflammatory and dysmetabolic activity, mainly due to alterations in insulin signaling and in the activity of FOXO1 and SREBP-1c factors (6, 21, 26).

### Cellular signaling pathways

Hepatokines exert their metabolic and hormonal effects through different intracellular signaling pathways, including PI3K-Akt, JAK-STAT, MAPK/ERK, AMPK and NF-κB (12, 26–28) (**Figure 1**).

The PI3K-Akt pathway is one of the key intracellular pathways upon which hepatokines act to exert their hormonal and metabolic actions on adipose tissue and muscle (27). This pathway is essential for insulin signaling by adjusting processes such as glucose uptake, glycogenosynthesis, and inhibition of gluconeogenesis. Its activation occurs upon binding of these hepatokines to tyrosine kinase receptors (such as FGFR, IGF-1R or the insulin receptor), which triggers the activation of PI3K and, subsequently, of Akt, generating the metabolic effects in the target tissues. Certain hepatokines, such as FGF21 and ANGPTL6, stimulate this intracellular metabolic pathway in adipose tissue and muscle, improving insulin sensitivity and favoring cellular glucose uptake. In contrast, fetuin-A inhibits this pathway by activating TLR4, contributing to the development of insulin resistance (12, 28).

The JAK-STAT pathway is activated mainly in adipose tissue by hepatokines such as IGF-1 and ANGPTL6, and to a lesser degree by FGF21 (12, 27). This pathway regulates processes such as cell growth, differentiation and immune response. Activation of the receptor induces autophosphorylation of JAKs, which in turn phosphorylate tyrosine residues in the receptor. These phosphorylated tyrosines serve as anchor sites for STAT (signal transducer and activator of transcription) proteins and its subsequent translocation to the nucleus to induce gene expression (12, 27).

The MAPK/ERK pathway regulates both cell proliferation and differentiation, in addition to the stress response. Certain hepatokines, such as FGF21, ANGPTL4, IGF-1 and fetuin-A, activate this pathway in adipose tissue and skeletal muscle, especially during fasting and exercise, favoring metabolic adaptation. Its activation leads to a chain of molecular signals in which proteins such as Ras, Raf, MEK and ERK participate, resulting in the activation of genes that regulate cell growth, survival and the maintenance of metabolic function (12, 26).

The AMPK pathway can be activated by hepatokines such as FGF21, ANGPTL4 and adropine, acting as an intracellular energy sensor (12). During metabolic stress or during physical exercise, AMPK is activated and promotes fatty acid oxidation, reducing lipogenesis and improving insulin sensitivity. All this is achieved by phosphorylation of energy metabolism enzymes (12, 26).

The NF-κB pathway participates in chronic inflammatory processes activated by hepatokines such as fetuin-A, SeP and ANGPTL4, especially associated with insulin and NAFLD (29). Its activation, induced by cellular stress or inflammatory signals, induces the entry of the NF-κB complex into the cell nucleus, activating proinflammatory genes. While Fetuin-A promotes this activation through the TLR4 receptor, aggravating inflammation and insulin resistance, FGF21 blocks this pathway in adipose tissue, exerting anti-inflammatory effects and improving insulin sensitivity (29–31).

## Functional classification

Although hepatokines are involved in different pathophysiological processes, they can be functionally classified according to their predominant functions (**Table 1**). This classification should not be considered rigid, as many of them have different effects which may vary according to the physiological or pathological context (6, 7).

**Table 1:** Functional classification of hepatokines according to their predominant physiological and pathophysiological effects.

Functional category	Main hepatokines	Key functions
Metabolic	FGF21, ANGPTL3, -4, -6, Adropin, SeP	Regulate glucose and lipid homeostasis; improve insulin sensitivity
Inflammatory/Immunomodulatory	Fetuin-A, SeP, ANGPTL4	Modulate immune responses; promote or regulate chronic inflammation
Regenerative	HGF, IGF-1, FGF21	Stimulate tissue repair and regeneration; support anabolic processes
Oncogenic or cancer-protective	Fetuin-A, ANGPTL4, IGF-1, FGF21	May promote or inhibit tumor progression depending on the physiological context

This functional classification of hepatokines is based on their predominant physiological or pathophysiological roles. Due to their pleiotropic nature, some hepatokines appear in more than one category depending on the biological context (e.g., metabolic stress, inflammation, or tumor microenvironment). ANGPTL4, Angiopoietin-like 4; ANGPTL6, Angiopoietin-like 6; FGF21, Fibroblast Growth Factor 21; Fetuin-A, Fetuin-A; HGF, Hepatocyte Growth Factor; IGF-1, Insulin-like Growth Factor 1; SeP, Selenoprotein P.

We can consider the following groups 1) Metabolic, such as FGF21, ANGPTL6, and SeP, which regulate glucose and lipid homeostasis, whose dysfunction is associated with metabolic diseases such as NAFLD and type 2 diabetes (10, 15); 2) Inflammatory or immunomodulatory, such as Fetuin-A, SeP and ANGPTL4, which are usually involved in chronic inflammatory processes related to metabolic diseases and participate in the immune response and hepatic inflammation, being relevant in the progression of nonalcoholic steatohepatitis (NASH) and other hepatopathies (32, 33); 3) Regenerative, such as HGF, IGF-1 and FGF21, which favor the repair and regeneration of damaged tissues (6, 7); and 4) Oncogenic or cancer-protective, such as Fetuin-A, ANGPTL4, IGF-1 and FGF21, depending on the biological environment in which they act (32, 33).

## Key hepatokines and their main physiological functions

**Figure 2:**
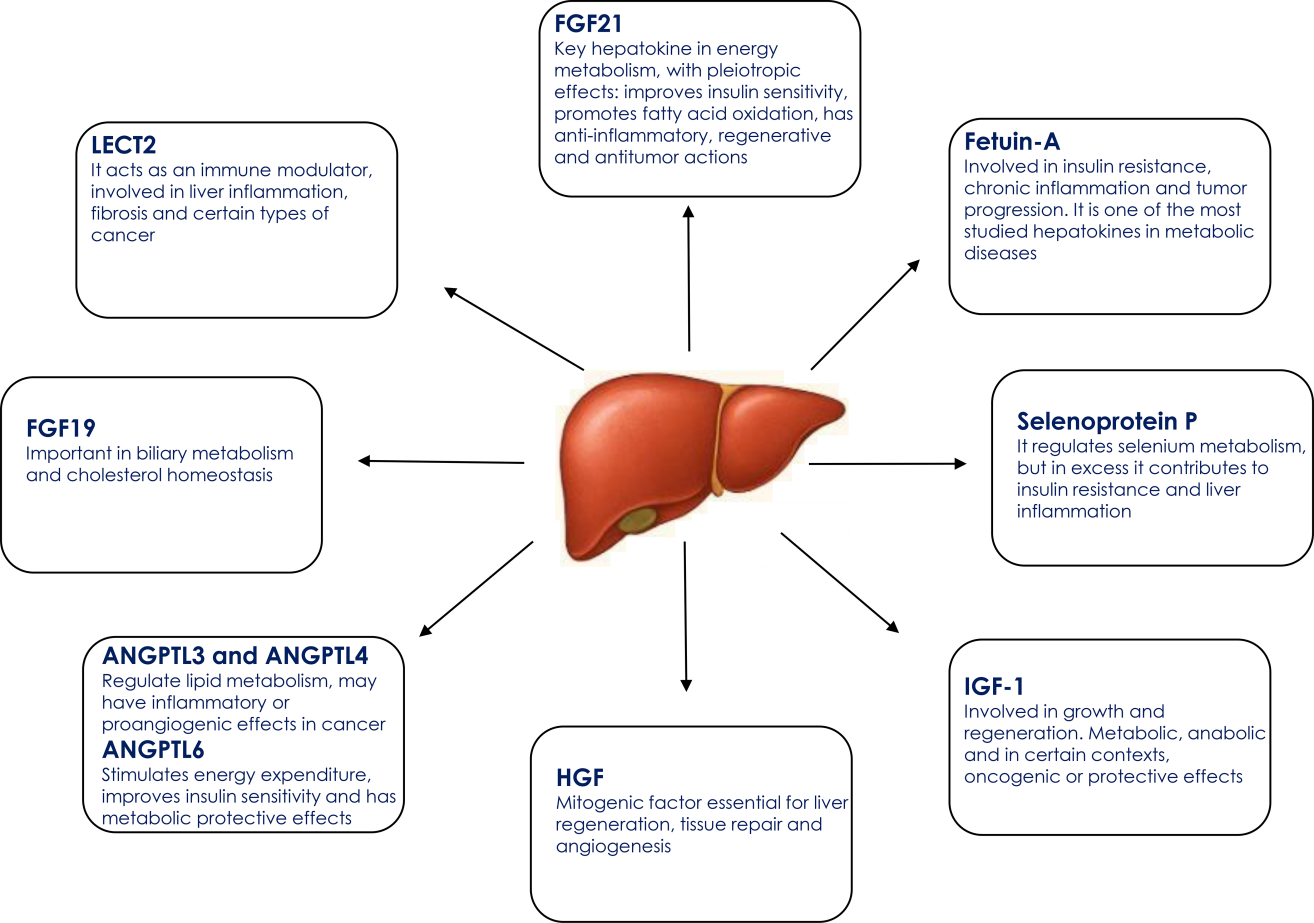
Key hepatokines and their main physiological functions. ANGPTL3, Angiopoietin-like protein 3; ANGPTL4, Angiopoietin-like protein 4; ANGPTL6, Angiopoietin-like protein 6; FGF19, Fibroblast growth factor 19; FGF21, Fibroblast growth factor 21; HGF, Hepatocyte growth factor; IGF-1, Insulin-like growth factor 1; LECT2, Leukocyte cell-derived chemotaxin 2.

Recent advances have identified several liver-derived proteins with systemic endocrine actions, modulating glucose metabolism, energy homeostasis, and inflammation through their effects on distant organs. **Figure 2** highlights the most extensively studied hepatokines described to date and summarizes their principal physiological functions.

### Fibroblast growth factor 21

Fibroblast growth factor (FGF21) was identified in 2000 by Nishimura et al. (34) as a member of the FGF family, although its metabolic role was described in more detail five years later by Kharitonenkov et al. (35). It is synthesized not only in the liver, but also in adipose tissue, the pancreas, and skeletal muscle. Its expression increases in response to fasting, ketogenic diet, exercise, protein restriction and through the activation of nuclear receptors such as PPAR-α in the liver and PPAR-γ in adipose tissue (13, 36, 37). FGF21 binds to its receptors FGFR1c and FGFR3c in the presence of the co-receptor β-Klotho. This hormone controls energy metabolism, increases insulin sensitivity, promotes fatty acid oxidation and ketogenesis during fasting, and prevents gluconeogenesis in the liver. It also improves glucose uptake in adipocytes and regulates energy consumption and body weight (13, 38–40).

### Fetuin-A

Fetuin-A, also known as α2-glycoprotein of Heremans-Schmid (AHSG) was discovered by Pedersen in 1944 (41), is an abundant glycoprotein in fetal serum, studied in depth in the 1970s. It is synthesized mainly in the liver, its expression is controlled by metabolic factors such as hyperglycemia and free fatty acids, in addition to inflammatory signals, and it decreases during acute inflammation and sepsis as it is a negative phase protein (42). Likewise, fetuin-A intervenes in calcium transport and helps to prevent ectopic calcification, although this protective role does not function well in the presence of metabolic alterations. It has also been related to cell proliferation processes and tumor progression. In summary, fetuin-A functions as a hepatokine with many functions, behavior strongly conditioned by the pathophysiological context (43). Levels are elevated in insulin resistance, obesity, non-alcoholic fatty liver disease (NAFLD) and metabolic syndrome.

### Selenoprotein P

Selenoprotein P (SeP), discovered in 1982 by Hill and Burk, is a glycoprotein synthesized mostly in the liver, encoded by the *SEPP1* gene (44, 45). It is the main protein of selenium transport in the blood, especially in the brain and testicles (46). Its expression depends on selenium levels, inflammation and liver function (47). In addition to its role in the transport of selenium, it serves as an antioxidant, and acts as a biomarker of body selenium status. SeP deficiency can affect neurological function and fertility. Its excess has been related to insulin resistance, endothelial dysfunction and increased risk of type 2 diabetes and cardiovascular disease (14, 48).

### Insulin-like growth factor 1

Insulin-like growth factor 1 (IGF-1) was identified in the 1950s by Salmon y Daughaday (49), although their sequence and structure were later characterized. It is a 70 amino acid peptide encoded by the IGF1 gene (12q23.2) and synthesized mainly in the liver under growth hormone (GH) stimulation, although it is also produced locally with autocrine and paracrine functions (50–53). IGF-1 expression, regulated by GH, functions through STAT5b. However, other factors also play a role such as insulin, nutritional status, thyroid hormones, inflammation, and age. IGFBP transporter proteins, especially IGFBP-3, control its half-life and also its bioavailability. IGF-1 mediates the anabolic effects of GH by promoting bone growth, cell proliferation, protein synthesis, energy metabolism, and neurogenesis as well as tissue repair. Its deficiency or excess is related to growth disorders, sarcopenia, and aging and, on the other hand, to cardiovascular or even neoplastic risk (53–55).

### Hepatocyte growth factor

Hepatocyte Growth Factor (HGF), discovered in 1984 by Toshikazu Nakamura et al, is a potent mitogen for hepatocytes in culture (56). It is synthesized primarily by mesenchymal cells such as fibroblasts, endothelial and vascular smooth muscle cells in organs such as liver, lung, kidney, bone marrow and adipose tissue. It is produced as an inactive precursor form (pro-HGF), which is activated by proteolysis by specific serine proteases such as HGF activator (HGFA) or urokinase-type plasminogen activator (uPA), generating a functional form of two chains linked by disulfide bridges (57–60).

HGF regulation occurs at both the transcriptional and post-translational levels, and its expression is markedly increased in response to tissue damage (hepatectomy, hepatitis, ischemia, inflammation or exposure to toxins). HGF expression is also regulated by different cytokines such as IL-1, IL-6, and TNF-α, growth factors, such as TGF-β and PDGF, hypoxia and different toxins. For example, carbon tetrachloride (CCl^4^) (61, 62) and lipopolysaccharide (LPS) (63, 64) are well-characterized hepatotoxins known to induce HGF expression during liver injury and regeneration. These agents act by triggering inflammatory responses and activating hepatic stellate cells.

Its receptor, c-MET, is a transmembrane tyrosine kinase through which HGF exerts pleiotropic effects, including, in addition to liver regeneration, renal repair, angiogenesis, neurogenesis, and immune modulation (60, 65). It also inhibits fibrosis and promotes regeneration of various tissues such as skeletal muscle and lung, and contributes to energy metabolism and insulin sensitivity (66, 67).

### Other hepatokines

In addition to the previously mentioned hepatokines, there are others that have metabolic and immunological functions. These include ANGPTL3 whose main function is to inhibit the enzymes lipoprotein lipase (LPL) and endothelial lipase, which limits the degradation of triglycerides and cholesterol-rich lipoproteins, thus raising plasma levels of triglycerides, LDL and HDL cholesterol (68); ANGPTL4, which participates in lipid metabolism by inhibiting lipoprotein lipase, and has been associated with proinflammatory and proangiogenic effects (69); and ANGPTL6 with metabolically protective properties by stimulating energy expenditure and improving insulin sensitivity (70).

Fibroblast growth factor 19 (FGF19) is essential for bile acid and cholesterol homeostasis. Its dysregulation has been associated with the pathogenesis of hepatocellular carcinoma (HCC) and, potentially, colorectal cancer, mainly through mechanisms related to cell proliferation and metabolic alteration (12, 71).

Leukocyte cell-derived chemotactin 2 (LECT2) has immunomodulatory functions in liver inflammation, fibrosis and HCC. It acts by promoting inflammation and fibrosis through macrophage activation and Tie1 receptor signaling, while, in HCC, it acts as a tumor suppressor by limiting progression, angiogenesis and accumulation of immunosuppressive cells (72, 73).

Among the most recently described hepatokines, adropine stands out for its capacity to regulate the selection of energy substrates and to favor metabolic flexibility according to nutritional status (74).

## Hepatokines in human disease

Alterations in the levels of various hepatokines have been described in multiple metabolic, inflammatory and neoplastic diseases. In addition to metabolic disorders, several previous studies have investigated the role of hepatokines in cardiovascular disease, cancer, autoimmune conditions, and tissue regeneration, highlighting their pleiotropic functions across different organ systems. These variations reflect their pathophysiological implication are summarized in **Table 2**.

**Table 2:** Summary of the main human hepatokines, their pathophysiological mechanisms and associated diseases.

Hepatokine	Pathophysiological mechanisms	Associated diseases	References
FGF21	Enhances fatty acid oxidation, insulin sensitivity, and glucose uptake; regulates energy expenditure and ketogenesis; resistance occurs in obesity.	Obesity, insulin resistance, dyslipidemia	(13, 36–39)
Fetuin-A	Activates TLR4 signaling; promotes inflammation and insulin resistance; involved in calcium transport and ectopic calcification; supports tumor progression.	Obesity, type 2 diabetes insulin resistance, NAFLD, metabolic syndrome	(41, 42)
Selenoprotein P	Transports selenium; excess induces oxidative stress, endothelial dysfunction, and reduced glucose uptake in muscle.	Type 2 diabetes, cardiovascular disease, insulin resistance	(44, 45)
IGF-1	Stimulates cell proliferation, protein synthesis, neurogenesis, and energy metabolism; imbalance leads to tissue dysfunction or overgrowth.	Sarcopenia, aging, cardiovascular disease, cancer	(53–55)
HGF	Promotes liver regeneration, angiogenesis, tissue repair, and insulin sensitivity; inhibits fibrosis and modulates immune response.	Liver disease, fibrosis, kidney injury, metabolic disorders	(56–59, 75)
ANGPTL3	Inhibits lipoprotein lipase and endothelial lipase, increasing triglycerides and cholesterol; modulates lipid metabolism; contributes to systemic inflammation and endothelial dysfunction	Familial hypercholesterolemia, mixed dyslipidemia, atherosclerosis, cardiovascular disease	(68)
ANGPTL4	Inhibits lipoprotein lipase, increasing triglycerides; promotes inflammation and angiogenesis.	Dyslipidemia, inflammation, vascular disease	(69)
ANGPTL6	Enhances energy expenditure and insulin signaling; suppresses hepatic gluconeogenesis.	Improves insulin sensitivity	(70)
FGF19	Regulates bile acid and cholesterol metabolism; dysregulation promotes proliferation and metabolic reprogramming.	Hepatocellular carcinoma, colorectal cancer	(12, 71)
LECT2	Promotes inflammation and fibrosis via macrophage M1 polarization and Tie1 signaling; acts as tumor suppressor in HCC.	NASH, liver fibrosis, hepatocellular carcinoma	(72, 73)
Adropin	Regulates energy substrate preference and enhances metabolic flexibility depending on nutritional status.	Insulin resistance, metabolic dysfunction	(74)

ANGPTL4, angiopoietin-like protein 4; ANGPTL6, angiopoietin-like protein 6; FGF19, fibroblast growth factor 19; FGF21, fibroblast growth factor 21; HGF, hepatocyte growth factor; IGF-1, insulin-like growth factor 1; LECT2, leukocyte cell-derived chemotaxin 2; NAFLD, non-alcoholic fatty liver disease; NASH, non-alcoholic steatohepatitis; TLR4, toll-like receptor 4.

### Metabolic diseases

Dysfunction of different hepatokines such as fetuin-A, LECT2, FGF21, ANGPTL6, SeP, and adropin alters liver-peripheral tissue communication favoring insulin resistance, dyslipidemia and chronic inflammation associated with obesity and metabolic síndrome (12). Similarly, alterations in various hepatokines are associated with the development of type 2 diabetes and insulin resistance. These include fetuin-A, SeP and LECT2 that promote insulin resistance, while others such as FGF21 and ANGPTL6 exert protective effects (6, 9, 76).

On the other hand, certain hepatokines have been involved in the pathophysiology and progression of NAFLD and its advanced form, NASH. These include FGF21, fetuin-A, SeP and ANGPTL4. Their secretion is altered in both entities, favoring insulin resistance, systemic inflammation, mitochondrial dysfunction and hepatic lipotoxicity, thus favoring the progression from simple steatosis to inflammation, hepatocellular damage and fibrosis (15, 77).

Although FGF21 has theoretically protective metabolic effects in these states its circulating concentrations are elevated reflecting functional resistance. Fetuin-A is associated with lipid accumulation and hepatic fibrosis, while SeP and ANGPTL4 enhance insulin resistance and hepatic inflammation. Although their measurement is not yet a clinical standard (78), hepatokines could be considered as potential future noninvasive biomarkers of disease progression and therapeutic targets in the management of NAFLD and NASH.

### Cardiovascular disease

Hepatokines also play a role in the pathophysiology of cardiovascular disease (CVD) due to their influence on metabolism and inflammation (79). Among those involved in this association are fetuin-A, FGF21 and ANGPTL4 as they are associated with insulin resistance, endothelial dysfunction, adverse cardiac remodeling and atherosclerosis, important factors in CV risk.

Fetuin-A promotes vascular inflammation via TLR4/NF-κB and is related to arterial calcification. Recently, the fetuin-A/adiponectin (F/A) ratio has been shown to correlate independently with subclinical atherosclerosis, as measured by carotid intima-media thickness (CIMT), and has greater predictive value than proteins alone in patients with newly diagnosed type 2 diabetes (80).

FGF21, despite its vasculoprotective effect, is usually elevated in altered metabolic states as a reflection of functional resistance, and its increase is linked to endothelial dysfunction and worse cardiac prognosis. A meta-analysis of 9 studies showed that elevated FGF21 levels were significantly associated with an increased risk of adverse CV events and mortality in patients with coronary artery disease (CAD), but showed no conclusive association in patients with heart failure (HF) due to high heterogeneity and publication bias (81). These findings position FGF21 as a possible prognostic biomarker in CAD, although its usefulness in HF still requires further evidence.

### Cancer

An association between some hepatokines and liver carcinogenesis has been described, particularly in the context of NAFLD/NASH, where they contribute to chronic inflammation, insulin resistance, fibrosis and tumor remodeling (32, 33).

NAFLD, particularly its progressive form NASH, is increasingly recognized as a major risk factor for the development of hepatocellular carcinoma (HCC). This progression occurs through a multistep process characterized by persistent insulin resistance, lipotoxicity, oxidative stress, and chronic inflammation, which contribute to hepatocellular injury, fibrosis, and eventually malignant transformation (82). Importantly, HCC in the context of NAFLD/NASH may develop even in the absence of cirrhosis (83, 84), distinguishing it from traditional viral- or alcohol-related liver cancers. In this pathological continuum, hepatokines play a key role by modulating inflammation, fibrogenesis, cell proliferation, and the immune microenvironment, thereby contributing to tumor initiation and progression (33). Their dual roles as both mediators and potential biomarkers of NAFLD-associated HCC highlight their clinical relevance in disease monitoring and therapeutic targeting.

IGF-1 is known to be associated with proliferative and anti-apoptotic effects. Several epidemiological studies and meta-analyses have shown a positive association between elevated IGF-1 levels and an increased risk of cancer, especially breast, prostate and colon cancer (85). These findings are corroborated by both classic cohort studies and recent large-scale cohort data demonstrating that higher circulating IGF-1 concentrations are associated with elevated cancer incidence, including breast and prostate cancer (86).

Insulin-like growth factor−1 (IGF−1) promotes tumor cell proliferation, migration, invasion, and immune evasion, and is associated with poor prognosis in multiple cancers (87). In gastric cancer, IGF−1 induces IFITM2 expression via IGF−1R/STAT3 signaling, thereby enhancing tumor growth and metastasis (88, 89). In melanoma, reducing IGF−1 levels suppresses cancer stem-cell characteristics and limits proliferation and metastatic behavior (90). In breast cancer cells, IGF−1 upregulates Cyr61 via PI3K/AKT, promoting growth and invasion (91). Furthermore, in HCC models, activation of IGF−1/IGF−1R signaling expands cancer stem cell (CSC) populations and enhances tumor growth and metastasis, while IGF−1R inhibition suppresses these effects both *in vitro* and *in vivo* (92). Moreover, in nasopharyngeal carcinoma models, osteoclast-derived IGF−1 activates AKT/S6 signaling and promotes bone metastasis; blocking IGF−1 significantly impairs metastatic colonization (93). From lifespan studies, GH/IGF−1–deficient mouse models (long-lived mutants) exhibit a reduced incidence and delayed onset of cancer, implicating diminished IGF−1 signaling in cancer resistance (94). Similarly, humans with Laron syndrome (GH insensitivity and low IGF−1) present a notably reduced cancer incidence, supporting the protective effect of reduced IGF−1 signaling (95). However, to date it is not advisable to use IGF-1 as a screening test in the general population since the relationship between IGF-1 and cancer is correlational and not causal, and its levels vary by non-oncological factors such as age, sex, nutritional status, exercise, among others. Finally, there is no evidence that its measurement improves diagnosis or prognosis, and it could also generate false positives (96–99).

Fetuin-A, FGF21, SeP and ANGPTL4 are related to cell proliferation, angiogenesis, metastasis and tumor immunosuppression (15, 100–102). Fetuin-A promotes tumor progression by activating PI3K/Akt and MAPK pathways, while in the context of hepatocarcinogenesis, FGF21 deficiency or dysfunction is associated with increased tumor proliferation and HCC progression. SeP and ANGPTL4 are involved in redox stress and metastatic invasion, respectively.

All these alterations make hepatokines potential biomarkers of tumor progression and possible future therapeutic targets, mainly in HCC as well as in systemic cancers related to metabolic dysfunction.

### Autoimmune diseases

Some hepatokines seem to be associated with different autoimmune diseases. Fetuin-A and chemerin are elevated in autoimmune diseases such as rheumatoid arthritis, lupus and psoriasis, correlating with inflammatory activity. On the other hand, FGF21 and adropin could play a protective role as they modulate the activation of T cells and monocytes (12, 27, 103, 104). Recent clinical data specifically link Fetuin-A dysregulation with psoriasis disease activity, reinforcing the immunometabolic relevance of hepatokines in systemic autoimmunity (105). These proteins could behave as immunometabolic biomarkers and potential therapeutic targets in the context of systemic inflammation and autoimmunity.

### Fibrosis and tissue repair

In addition, hepatokines such as HGF have been extensively investigated for their role in tissue regeneration, modulation of fibrosis, and immune regulation; HGF promotes repair processes across liver, lung, kidney, and skeletal muscle, while attenuating fibrosis and improving insulin sensitivity—highlighting its therapeutic potential beyond metabolism (66, 106).

## Potential clinical and therapeutic applications

Although there are currently no approved therapies based directly on hepatokines, their study is advancing as part of precision medicine, with possible diagnostic, prognostic and therapeutic applications in different systemic diseases.

The potential clinical and therapeutic applications of hepatokines are focused on metabolic, hepatic and cardiovascular diseases. Some of them such as FGF21, fetuin-A, SeP and ANGPTL8 are investigated as biomarkers to detect and monitor insulin resistance, dyslipidemia, type 2 diabetes, NAFLD/NASH and cardiovascular risk (15, 107–109).

Modulation of proinflammatory hepatokines such as fetuin-A and SeP represents a promising field, but still in the translational research phase.

Normalization of IGF-1 levels in patients with acromegaly by surgery, drug treatment or radiotherapy is associated with a reduction in cancer risk. However, a slightly elevated residual risk may persist, probably due to previous prolonged exposure to high IGF-1 and GH levels (110, 111). In contrast, in cases of GH deficiency, increasing IGF-1 to physiological levels does not appear to increase the risk of cancer, as long as it remains within normal ranges (112).

FGF21 analogs have been positioned as potential candidates for the treatment of metabolic diseases such as type 2 diabetes, obesity and NASH. Currently, several compounds are in advanced stages of clinical development, most notably Efruxifermin (Akero Therapeutics) and BIO89-100 (89bio, (pegozafermin) due to their favorable efficacy and safety profile (113, 114).

In the phase 2a BALANCED Efruxifermin (Fc-FGF21 analog) study, treatment with efruxifermin for 16 weeks in patients with NASH (stage F1-F3) was associated with resolution of NASH without worsening of fibrosis in approximately 76% of patients, accompanied by 5-8% weight loss, reduced HbA1c and improved lipid profile, with mild to moderate gastrointestinal adverse effects being the most common (115). In the phase 2b HARMONY trial, which included patients with NASH and moderate to advanced fibrosis (F2-F3), weekly administration of efruxifermin (28 or 50 mg) for 24 weeks achieved significant histological improvement. The treatment was generally well tolerated, with mild to moderate gastrointestinal adverse effects being the most frequent (116). In patients with metabolic dysfunction-associated steatohepatitis (MASH), fibrosis and type 2 diabetes treated with GLP-1 receptor agonists (GLP-1Ras), efruxifermin for 12 weeks significantly reduced (65%) hepatic fat and improved noninvasive markers of fibrosis and metabolism, suggesting additional hepatic benefits of combining efruxifermin with GLP-1RAs in this population (113). However, in patients with compensated cirrhosis caused by MASH, efruxifermin did not significantly reduce fibrosis at 36 weeks (117).

BIO89-100, a novel glycoPEGylated analog of FGF21, improved triglyceride levels, cholesterol, glucose metabolism, adiponectin levels and liver enzymes in both diabetic monkeys and healthy humans (118). A phase 2b trial in patients with biopsy-confirmed NASH demonstrated that weekly or biweekly doses of pegozafermin produced significant improvements in liver fibrosis and resolution of NASH compared to placebo (119). On the other hand, this analog was evaluated in a randomized, double-blind, placebo-controlled phase 2 clinical trial in patients with severe hypertriglyceridemia. The treatment significantly reduced triglycerides by 57.3% vs. 11.9% placebo, non-HDL cholesterol by 18.3% vs. 0.6%, and liver fat by 42.2% vs. 8.3%, and showed a good safety profile (120).

ANGPTL3 inhibitors are aimed at reducing blood lipid levels, especially in patients with severe dyslipidemias or cardiovascular disease. Blockade of ANGPTL3, a protein that inhibits the enzymes LPL and endothelial lipase, both essential for the degradation of triglycerides and cholesterol, is accompanied by a reduction in triglycerides, total cholesterol, LDL cholesterol and, to a lesser extent, HDL cholesterol (121).

One of the most studied inhibitors is evinacumab, a monoclonal antibody against ANGPTL3, which has shown high efficacy in reducing LDL cholesterol levels, even in patients with homozygous familial hypercholesterolemia (HoFH), even in those resistant to conventional treatment (122–126). Phase 3 clinical trials showed that, in patients with HoFH, evinacumab reduced LDL-C by 47%, while placebo increased it by 2%, with good tolerability (124).

Other approaches are also being developed, including RNA-based therapies such as antisense oligonucleotides directed against ANGPTL3 through the inhibition of hepatic ANGPTL3 mRNA translation, which reduces the synthesis of the protein and consequently decreases its plasma concentration. Multiple doses reduced ANGPTL3 by 46.6-84.5%, triglycerides by up to 63.1%, LDL cholesterol by up to 32.9%, VLDL by up to 60%, non-HDL cholesterol by up to 36.6%, ApoB by up to 25.7% and ApoC-III by up to 58.8%, without serious adverse effects (127).

## Current limitations and challenges in its clinical use

One of the main challenges in the study of hepatokines lies in the complexity of their biological regulation and the functional overlap they present with other cytokines and hormones, which makes it difficult to identify precise therapeutic targets. In addition, much of the current knowledge comes from animal models, whose applicability to humans is limited due to important physiological differences. In the field of clinical research, additional obstacles persist, such as the heterogeneity of human studies and the lack of standardization in the measurement of these molecules, which complicates the comparison of results and limits the robustness of the conclusions. Therefore, longitudinal studies in the human population and well-structured clinical trials are essential to clarify more precisely the role of these molecules in different metabolic pathologies. A deeper understanding of their mechanisms of action, their interactions and their effects at the systemic level will be key to assessing their potential usefulness in the diagnosis or treatment of these diseases in the future.

## Conclusions

Hepatokines are involved in different functions involved in the regulation of lipid and glucose metabolism, as well as in the inflammatory response. Their involvement in the onset and progression of metabolic diseases such as type 2 diabetes, dyslipidemia and NAFLD has led to an increasing interest in their study. It is important to know their mechanisms of action in order to identify specific biomarkers and develop targeted therapies, within the context of personalized medicine.
